# Cutaneous Squamous Cell Carcinoma with Signet-Ring Cell Component and CDX2 Expression in a Patient Treated with PD-1 Inhibitor: A Case Report of a Common Tumor with Unusual Differentiation

**DOI:** 10.1155/2023/3378044

**Published:** 2023-02-09

**Authors:** Sherehan Zada, Jeremiah Tao, Maria Del Valle Estopinal

**Affiliations:** ^1^Ophthalmic Pathology Division, Department of Pathology and Laboratory Medicine, University of California Irvine, USA; ^2^Gavin Herbert Eye Institute, University of California Irvine, USA

## Abstract

Signet-ring cell squamous cell carcinoma (SRCSCC) is an uncommon variant of cutaneous SCC that has been reported in the head and neck region. Herein, we present a case of a 56-year-old female with a cutaneous SCC that recurred after surgical excision, during treatment with cemiplimab (a programmed death receptor-1 (PD-1) inhibitor). Histologically, the recurrent SCC revealed a second component characterized by the presence of signet-ring-like cells (SRLCs). Immunohistochemical studies demonstrated that the tumor cells were positive for P63, CK5/6, CDX2, and P53 while negative for P16, CK7, CK20, and CD68 stains. An abnormal expression of B-catenin was also observed in the tumor. To our knowledge, SRCSCC developing during treatment with an immune checkpoint inhibitor has not been documented in the literature. Our findings suggest a form of acquired SCC cell resistance to immunotherapy that might involve CDX2-related pathways.

## 1. Introduction

Squamous cell carcinoma (SCC) is the second most common type of skin cancer. It usually originates from the epidermal keratinocytes with variable degrees of differentiation and cytologic atypia [1, 2]. SCC with signet-ring cell morphology is extremely uncommon and has been reported in the head and neck region. Signet-ring cells are associated with adenocarcinomas and a more aggressive behavior; however, the mechanisms involved in signet-ring cell formation in other tumors are uncertain [3, 4]. We describe, for the first time to our knowledge, a cutaneous SRCSCC of the midforehead that developed during systemic PD-1 inhibitor therapy for a separate unresectable eyelid SCC with secondary orbital invasion.

## 2. Case Presentation

**Figure 1:**
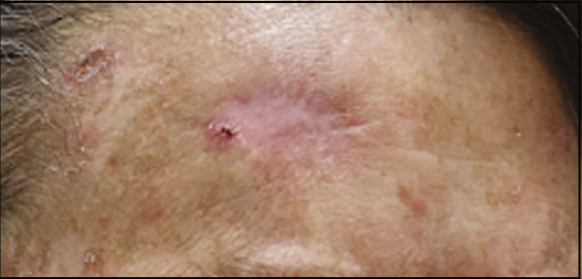
Clinical picture of the midforehead depicting an erythematous scaling papule in a background of atrophic scar.

A 56-year-old female with a history of multiple periocular and midforehead SCCs and basal cell carcinomas (BCCs) presented with a new midforehead lesion arising in a prior resection site that proved to be a moderately differentiated cutaneous SCC. She was on treatment with cemiplimab (PD-1 inhibitor), 8 cycles administered over 1 year, for an unresectable left eyelid SCC that had invaded the anterior and midinferior left orbit. This orbital mass had regressed favorably with the PD-1 inhibitor therapy. The patient's clinical history was negative for any other visceral malignancy, radiation exposure, or family history of skin malignancy. On examination, an erythematous scaly papule was identified above the right eyebrow, measuring 0.4 cm in a background of hypopigmented, slightly atrophic scar (Figure 1). The clinical differential diagnosis included recurrent SCC and actinic keratosis, among other lesions.

**Figure 2:**
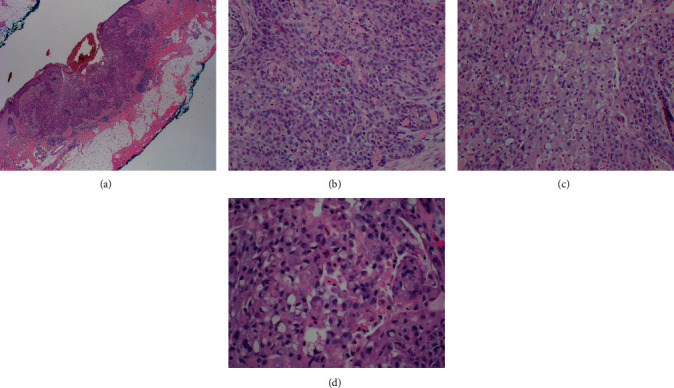
Histopathologic analysis. (a) Superficial skin ulcer associated with lobules and strands of atypical squamous cells infiltrating the dermis. Chronic inflammatory infiltrate is noted at the base of the tumor. H&E (original magnification ×20). (b) Moderate to poorly differentiated squamous cells with minimal keratinization are observed. H&E (original magnification ×200). (c) Tumor depicting squamous and signet-ring-like cell components. H&E (original magnification ×200). (d) Signet-ring-like cells displaying displaced nuclei with clear cytoplasm and occasional eosinophilic cytoplasmic inclusions. H&E (original magnification ×400).

Microscopically, the skin excisional biopsy demonstrated a central superficial ulcer associated with lobules and strands of atypical keratinocytes infiltrating the underlying dermis. Lobules of polyhedral neoplastic cells were focally connected to the epidermis and characterized by vesicular nuclei with large central nucleolus and scant eosinophilic cytoplasm. Approximately 30% of the tumor was composed of acantholytic and dyskeratotic cells admixed with SRLCs. A moderate lymphocytic infiltrate was observed along the base of the tumor (Figure 2). There was a background of actinic keratosis and focal dermal scar adjacent to the tumor.

Perineural invasion was identified, but there was no definite evidence of lymphovascular invasion.

**Figure 3:**
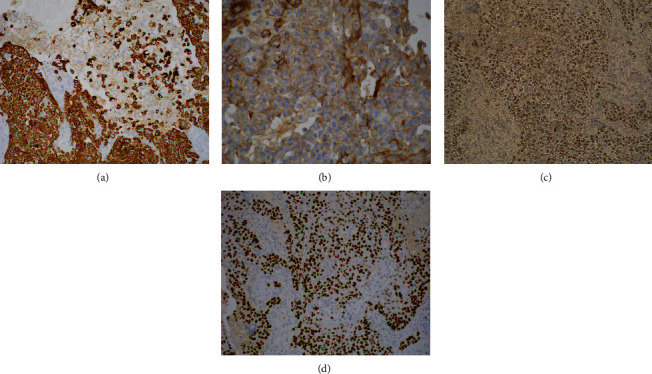
Immunohistochemical findings: (a) CK5/6 stain depicting positive staining in the squamous and the signet ring-like cells (original magnification ×200); (b) beta-catenin stain showing weak and incomplete membranous staining in tumor cells (original magnification ×400); (c) CDX2 stain revealing weak to moderate positive nuclear staining in the tumor (original magnification ×400); (d) P63 stain highlighting nuclei of tumor cells (original magnification ×200).

Intracytoplasmic eosinophilic globules were noted in the SRLCs, highlighted with periodic acid-Schiff (PAS), but negative for mucicarmine stain. These findings suggested that the cytoplasmic deposits were derived from glycogen. Formalin-fixed, paraffin embedded 4-micron unstained tissue sections were analyzed immunohistochemically with the following monoclonal antibodies: p63 (4A4, RTU, Ventana/Roche, Santa Clara, CA, USA), cytokeratin 5/6 (CK5/6) (D5/16B4, RTU, Ventana/Roche, Santa Clara, CA, USA), p53 (D0-7, 1 : 400, Dako, Carpinteria, CA, USA), caudal-type homeobox transcription factor 2 (CDX2) (EPR2764Y, RTU, Cell Marque, Rocklin, CA, USA), p16 (E684, TRU, Ventana/Roche, Santa Clara, CA, USA), cytokeratin 7 (CK7) (SP52, RTU, Ventana/Roche, Santa Clara, CA, USA), cytokeratin 20 (CK20) (SP33, RTU, Ventana/Roche, Santa Clara, CA, USA), CD68 (KP-1, RTU, Ventana/Roche, Santa Clara, CA, USA), and beta-catenin (14, RTU, Ventana/Roche, Santa Clara, CA, USA). All antibodies and testing were performed in a Clinical Laboratory Improvement Amendments (CLIA)-certified laboratory with standardized clinical methodology and positive control tissue. The immunohistochemical studies revealed two types of tumor cells positive for P63, CK5/6, and P53 (positive in 35-40% of tumor cells) and weak to moderately positive for CDX2 (Figures 3(a), 3(c), and 3(d)) while negative for P16, CK7, CK20, and CD68 stains. Heterogeneous, incomplete membranous staining for beta-catenin was observed in more than 50% of tumor cells (Figure 3(b)).

The SRLC component was not identified on the histopathologic sections of the prior SCC skin excision, obtained 5 months earlier. The presence of this new component in the re-excised SCC tumor, at the same location of prior tumor, supported the diagnosis of recurrent cutaneous SCC with SRLC component.

Follow-up examination, 12 months later, demonstrated a recurrent BCC in the temporal area and an unremarkable scar at the previous SCC site.

## 3. Discussion

SCC is a tumor of keratinocytes that is considered the second most common malignancy of keratinocyte carcinomas worldwide and accounts for 20-50% of all skin cancers [1]. SCC may show different histologic variants including basaloid, adenosquamous, papillary, and sarcomatous, among others [2]. SRCSCC is extremely uncommon and usually documented in the head/neck region [3–5]. Herein, we are reporting an additional case and the first in the setting of PD-1 inhibitor treatment.

The term signet-ring cell has been used to describe cells with abundant mucin accumulation and subsequent peripheral displacement of the nucleus [3]. This terminology has been frequently reported in carcinomas with glandular differentiation affecting predominantly the gastrointestinal tract, breast, bladder, pancreas, and lungs [3, 4]. Later, this pattern has been associated with a variety of skin and nonskin neoplasms including liposarcomas, melanomas, BCCs, SCCs, and lymphomas [3]. The exact etiology of this phenomenon is not completely understood; however, glycogen accumulation, lipid accumulation, keratin, or cytoplasmic hydropic changes are all possible suggested theories [3].

Variable risk factors have been involved in the pathogenesis of SCC including light skin type, ultraviolet sun exposure, human papillomavirus (HPV), immunosuppression, and environmental exposure. Additionally, numerous gene mutations have been associated with SCC pathogenesis including tumor protein *p53* gene, cyclin dependent-kinase inhibitor 2A, and RAS proteins [1]. The SRC variant has demonstrated an association with ultraviolet exposure. The role of HPV infection in the development of this morphology has also been considered with many other pathways that are still under investigation [4]. The prognostic significance of this morphology is not completely clear; however, some studies have documented a more aggressive behavior among this variant [3–5].

Dysregulation of Wnt signaling, including Wnt/B-catenin and B-catenin-independent pathways, has also been described as an important cofactor in the development, progression, and invasion of keratinocyte carcinomas. Wnt signaling can be stimulated by different Wnt ligand-receptor combinations. This pathway plays a significant role in cell proliferation, passage, and polarity. Accordingly, any deviation in this pathway may cause serious pathogenesis including cancer [6].

Beta-catenin protein plays an important role in the Wnt signaling pathway and hair morphogenesis. Normal beta-catenin expression is characterized by a homogeneous membranous staining along the intercellular junctions of the epidermis while a mutant protein is expressed in the nucleus [7]. An abnormal staining for beta-catenin has been reported in cutaneous carcinomas including SRC/histiocytoid carcinoma of the eyelid, BCC, and matrical carcinomas, among others [7, 8]. Fedeles et al. [7] described two cases of panfollicular carcinoma which displayed an aberrant nuclear and cytoplasmic expression of beta-catenin in tumor cells. Our findings are similar to those previously described in primary SRC/histiocytoid carcinoma of the eyelid in which an incomplete, heterogenous cytoplasmic/membranous staining was noted in more than 10% but less than 90% of tumor cells [8].

CDX2 protein is a transcriptional factor that plays an essential role in the development of the intestine. CDX2 nuclear expression has been reported in the colon, rectum, small intestine, and in more than 90% of colorectal adenocarcinomas including the SRC carcinoma variant of the gastrointestinal tract [9]. Recently, CDX2 expression has been described in pilomatrical tumors. Tumminello and Hosler [10] propose that CDX2 expression could play a role in the activation of the Wnt signaling pathway and dysregulation of beta-catenin and T cell factor/lymphoid enhancer-binding factor 1 (TCF-LEF-1) pathways in tumors arising from the germinative matrix of hair follicles which suggest similarities in the pathogenesis of colorectal carcinoma and pilomatrix carcinoma [10]. CDX2 expression in signet-ring cell carcinoma of the gastrointestinal tract can be variable. Chu and Weiss [11] have documented heterogenous weak patchy nuclear staining in gastric signet-ring cell carcinomas comparing to intense diffuse nuclear staining in colon signet-ring cell carcinoma. However, the significance of these patterns is unknown.

The use of cemiplimab to treat SCC has been approved by the Food and Drug Administration (FDA) since 2018 [12]. PD-1/PDL-1 (programmed death ligand 1) inhibitors are a class of immune checkpoint inhibitors (ICIs) that have been used frequently in treating unresectable SCCs with variable responses [13]. The PD-1/PDL-1 axis inhibits immune response reducing T cell activation, proliferation, survival, and cytokine secretion within the tumor environment. Additionally, expression of PDL-1 receptors has been documented in tumor cells as an adaptive immune mechanism. Therefore, the blockage of this pathway using checkpoint inhibitors will work against cancer cells [14]. Some SCCs show elevated levels of expression which has been associated with a more aggressive course and higher recurrence rates. The expression of programmed death PD-1 and PDL-1 in SCC gives the advantage of using PD-1/PDL-1 inhibitors in the treatment of these tumors [13, 14]. The side effects of this therapy may include diarrhea, fatigue, nausea, constipation, and rash, among others [14].

Variable response to immune check point inhibitors has been recorded in the literature, and resistance to ICIs is a concern for this treatment. Although the underlying mechanisms continue under the scope of researchers, primary resistance or no response to the treatment occurs as a result of insufficient tumor immunogenicity, major histocompatibility complex dysfunction, irreversible T cell exhaustion, and/or immunosuppressive microenvironment, amid other pathways [14, 15]. On the other hand, tumor cells can avoid antitumor immunity during treatment due to genomic alteration, elevating PD-L1 expression, and/or reexhaustation of T cells, leading to an acquired resistance [15]. Tumor resistance has been documented through clinical progression and radiologic findings [14]. However, the histologic features of tumor resistance have not been described in the literature due to infrequent tumor sampling during treatment.

## 4. Conclusion

We describe a case of recurrent cutaneous SCC with a new signet-ring-like cell component that is diagnosed during immunotherapy. The recurrence of SCC with a more aggressive histologic features during ICI administration might have occurred by chance. However, the possibility that this rare morphology represents an adaptive mechanism of the tumor cells to survive therapy-related changes cannot be entirely excluded. Additionally, the expression of CDX2 and the weak, heterogeneous pattern of staining for beta-catenin in the tumor cells could represent abnormalities in the Wnt signaling pathway that have also participated in the development of this uncommon differentiation. Further studies should be considered to elucidate the histopathologic findings of tumor resistance to ICIs and the associated interconnecting molecular pathways in the pathogenesis of SCC.

## References

[1] Que S., Zwald F. O., Schmults C. (2018). Cutaneous squamous cell carcinoma: Incidence, risk factors, diagnosis, and staging. *Journal of the American Academy of Dermatology*.

[2] Diaz-Cascajo C., Borghi S., Weyers W., Bastida-Inarrea J. (2004). Follicular squamous cell carcinoma of the skin: a poorly recognized neoplasm arising from the wall of hair follicles. *Journal of Cutaneous Pathology*.

[3] Lortscher D. N., Satter E. K., Romero L. S. (2012). Signet ring-like cells: no longer a “Signature” of glandular differentiation. *Dermatology Online Journal*.

[4] Wang N. R., Wang M. M., Zhou L. (2016). Cutaneous clear cell/signet-ring cell squamous cell carcinoma arising in the right thigh of a patient with type 2 diabetes: combined morphologic, immunohistochemical, and etiologic analysis. *Diagnostic Pathology*.

[5] Findeis S. K., Readinger A., Mitchell J., Agarwal A. (2020). Cutaneous signet-ring cell squamous cell carcinoma. *Baylor University Medical Center Proceedings*.

[6] Lang C. M. R., Chan C. K., Veltri A., Lien W. H. (2019). Wnt signaling pathways in keratinocyte carcinomas. *Cancers*.

[7] Fedeles F., Cha J., Chaump M. (2013). Panfollicular carcinoma or trichoblastic carcinoma with panfollicular differentiation?. *Journal of Cutaneous Pathology*.

[8] Estopinal M. D. V., Middleton L. P., Esmaeli B., Patel K. P., Nowroozizadeh S., Williams M. D. (2021). Primary signet ring cell/histiocytoid carcinoma of the eyelid: clinicopathologic analysis with evaluation of the E-cadherin/*β*-catenin complex and associated genetic alterations. *Case Reports in Pathology*.

[9] Saad R. S., Ghorab Z., Khalifa M. A., Xu M. (2011). CDX2 as a marker for intestinal differentiation: its utility and limitations. *World Journal of Gastrointestinal Surgery*.

[10] Tumminello K., Hosler G. A. (2018). CDX2 and LEF-1 expression in pilomatrical tumors and their utility in the diagnosis of pilomatrical carcinoma. *Journal of Cutaneous Pathology*.

[11] Chu P. G., Weiss L. M. (2004). Immunohistochemical characterization of signet-ring cell carcinomas of the stomach, breast, and colon. *American Journal of Clinical Pathology*.

[12] Keeping S., Xu Y., Chen C. I. (2021). Comparative efficacy of cemiplimab versus other systemic treatments for advanced cutaneous squamous cell carcinoma. *Future Oncology*.

[13] Pezeshki S., Hemmati S., Rezaei N. (2020). Novel treatments using PD1 inhibitors for advanced and metastatic cutaneous squamous cell carcinoma. *Expert Review of Anticancer Therapy*.

[14] Migden M., Rischin D., Schmults C. D. (2018). PD-1 blockade with cemiplimab in Advanced Cutaneous Squamous-Cell carcinoma. *The New England Journal of Medicine*.

[15] Lei Q., Wang D., Sun K., Wang L., Zhang Y. (2020). Resistance mechanisms of anti-PD1/PDL1 therapy in solid tumors. *Frontiers in Cell and Development Biology*.

